# CRISPR-based diagnostics of different biomolecules from nucleic acids, proteins, and small molecules to exosomes: CRISPR-based diagnostics of different biomolecules

**DOI:** 10.3724/abbs.2023134

**Published:** 2023-08-01

**Authors:** Yuanshou Zhu, Meng Zhang, Shujuan Guo, Hong Xu, Zhijun Jie, Sheng-ce Tao

**Affiliations:** 1 Shanghai Center for Systems Biomedicine Key Laboratory of Systems Biomedicine (Ministry of Education) Shanghai Jiao Tong University Shanghai 200240 China; 2 School of Biomedical Engineering Med-X Research Institute Shanghai Jiao Tong University Shanghai 200030 China; 3 Department of Pulmonary and Critical Care Medicine Shanghai Fifth People’s Hospital Fudan University Shanghai 200240 China; 4 Center of Community-Based Health Research Fudan University Shanghai 200240 China

**Keywords:** CRISPR/Cas, molecular diagnostics, biomolecule detection, point-of-care testing

## Abstract

CRISPR-based detection technologies have been widely explored for molecular diagnostics. However, the challenge lies in converting the signal of different biomolecules, such as nucleic acids, proteins, small molecules, exosomes, and ions, into a CRISPR-based nucleic acid detection signal. Understanding the detection of different biomolecules using CRISPR technology can aid in the development of practical and promising detection approaches. Unfortunately, existing reviews rarely provide an overview of CRISPR-based molecular diagnostics from the perspective of different biomolecules. Herein, we first introduce the principles and characteristics of various CRISPR nucleases for molecular diagnostics. Then, we focus on summarizing and evaluating the latest advancements in CRISPR-based detection of different biomolecules. Through a comparison of different methods of amplification and signal readout, we discuss how general detection methods can be integrated with CRISPR. Finally, we conclude by identifying opportunities for the improvement of CRISPR in quantitative, amplification-free, multiplex, all-in-one, and point-of-care testing (POCT) purposes.

## Introduction

Recurring pandemics caused by emerging viruses such as SARS, MERS, Ebola, Zika, and SARS-CoV-2 have made it necessary to develop quick response and point-of-care testing (POCT) methods [1]. The current gold standard detection method, PCR, which requires a central laboratory and has a long turnaround time, is inadequate for these requirements. CRISPR (clustered regularly interspaced short palindromic repeats) has emerged as a promising detection technology in the field of molecular diagnostics due to its easy programmable design, near-ambient reaction temperature, and high specificity and sensitivity for mutation identification [ 2‒ 5]. Although many reviews discussing CRISPR diagnostics have been published, they usually focus on nucleic acid detection and rarely summarize the latest developments from the perspective of different biomolecules, including nucleic acids, proteins, small molecules, exosomes, ions, and more. This review aims to fill this gap.

Originally, CRISPR was a prokaryotic adaptive immune system applied to resist virus and plasmid invasion in bacteria and archaea [6]. However, since the first detection method based on CRISPR/Cas9 was reported for Zika virus detection in 2016 [7], CRISPR-based molecular diagnostics have flourished, largely due to the discovery of the *cis*- and *trans*-cleavage ability of Cas12a, Cas12b, Cas13a, and Cas14a proteins [ 8‒ 12]. These Cas proteins can *trans*-cleave single-strand reporters after specifically *cis*-cleaving the target region matched with crRNA (CRISPR RNA). Both binding and cleavage of the target region by the CRISPR/Cas system require recognition of a short trinucleotide protospacer adjacent motif (PAM), which is commonly composed of a direct repeat region and a spacer region [13]. Taking Cas12a as an example, its canonical PAM is TTTN. However, Lu *et al* . [14] recently reported that suboptimal PAMs (NTTV and TTNT) showed better performance than canonical PAMs in a one-pot reaction of recombinase polymerase amplification (RPA) and Cas12a detection [14]. This indicated that suboptimal PAMs slow the kinetics of Cas12a-mediated *cis*-cleavage of substrates and *trans*-cleavage of fluorescent reporters, which leads to stronger fluorescence owing to the accumulation of amplicons generated by RPA. The flexibility of PAMs broadens the scope of crRNA design and improves the generality of CRISPR. By designing specific crRNA and adding the corresponding Cas proteins, target sequences, and single-strand reporters into the detection system, the Cas proteins will bind to crRNA to form the binary complex Cas/crRNA and then bind to the target sequences to form the ternary complex Cas/crRNA/target, which will *trans*-cleave nontargeted reporters and release signals. Additionally, dCas9, a variant of Cas9 that only binds to target DNA but cannot cleave it, is also applied in molecular diagnostics [ 15‒ 18]. Through a comparison of the Cas proteins used in the detection of different biomolecules, this review hopes to provide insights into how to combine the CRISPR system with other techniques in the future.

**Figure 1:**
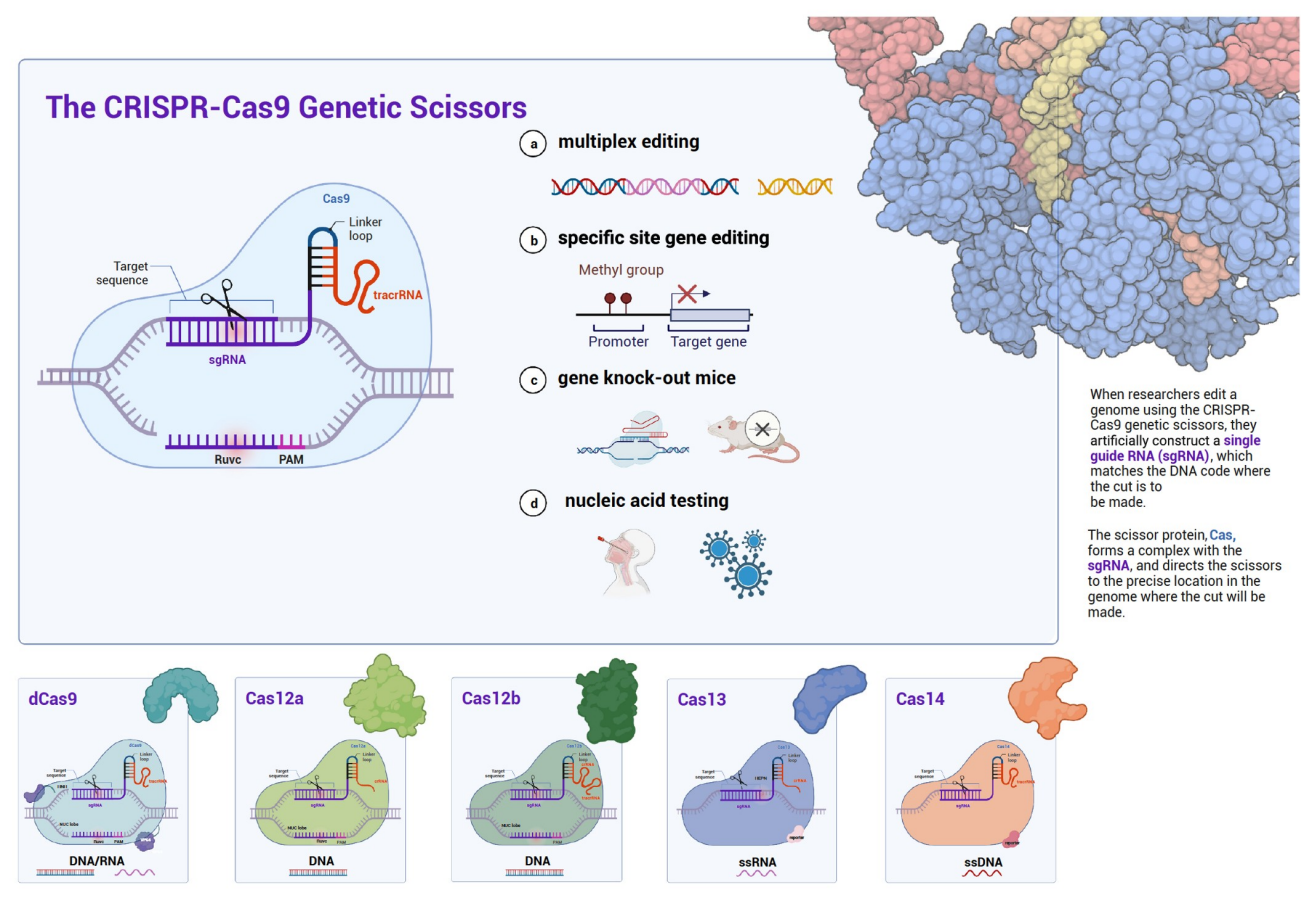
Figure 1 Illustration of the principles of several Cas proteins applied for molecular diagnostics Apart from the applications of multiplex editing, specific site gene editing, and gene knock-out, CRISPR-Cas9 system has been applied for nucleic acid testing. The current commonly used Cas proteins for molecular diagnostics also include dCas9, Cas12a, Cas12b, Cas13, and Cas14. This figure was created with BioRender.com.

This review first illustrates the principles of several Cas proteins applied in molecular diagnostics ( Figure 1). Then, we systematically summarize the current developments in CRISPR-based detection of different biomolecules. In particular, the review outlines the general process of combining CRISPR with other techniques from three aspects: sample preamplification, CRISPR detection, and signal readout (including signal amplification based on electronic and biochemical sensors). Finally, the review provides a comparison of the advantages and disadvantages of some mainstream CRISPR-based detection methods ( Table 1) and concludes by pointing out future directions for the development of CRISPR-based molecular diagnostics.

**Table:** **Table 1** The comparison of the mainstream CRISPR-based detection technologies mentioned in this article

Method	Target	Pre-amplification	Cas protein	Signal readout	Sensitivity	Time (min)	Ref.
NASBACC	RNA	NASBA	Cas9	Colorimetric	3 fM	180	[7]
CRISPR chip	DNA	N	dCas9	Electronic sensor	1.7 fM	15	[18]
SHERLOCK	NA	RPA	Cas13a	Fluorescence	2 aM	60	[19]
SHERLOCKv2	NA	RPA	Cas13a, Cas13b, Cas12a	Fluorescence, LFA	8×10 ^‒3^ aM	60	[20]
DETECTR	DNA	RPA	Cas12a	Fluorescence	2 aM	70	[3]
Cas14a-DETECTR	ssDNA	PCR	Cas14a	Fluorescence	2 aM	120	[12]
HOLMES	DNA	PCR	Cas12a	Fluorescence	10 aM	60	[21]
HOLMESv2	NA	LAMP	Cas12b	Fluorescence	10 ^‒8^ nM	60	[11]
mCARMEN	NA	RPA	Cas13a	Digital fluorescence	2 aM	60	[22]
opvCRISPR	RNA	LAMP	Cas12a	Fluorescence	5 copies	45	[23]
MEDICA	NA	RPA	Cas13a	Digital fluorescence	5 copies/μL	10‒25	[24]
AIOD-CRISPR	RNA	RPA	Cas12a	Fluorescence	3 copies	40	[25]
miSHERLOCK	NA	RPA	Cas13a	Fluorescence	1000 copies/mL	55	[26]
UCAD	Antibody	RPA	Cas12a	Fluorescence	10 aM	40	[27]
CLISA	IL-6, VEGF	T7 transcription	Cas13a	Fluorescence	2.29 fM, 0.81 fM	30	[28]
CaT-SMelor	Uric acid, p-HBA	N	Cas12a	Fluorescence	10 nM, 1.8 nM	15‒25	[29]
CaT-SMelor v2	AFP, cocaine	N	Cas12a	Fluorescence	0.07 fM, 0.34 μM	18	[30]
iPCCA	IL-6	N	Cas12a	Fluorescence	1 fM, 100 fM	120	[31]
MFS-CRISPR	Exosome	N	Cas13a	Fluorescence	1200 particles/mL	60	[32]
ARTCA	Exosome	RPA	Cas13a	Fluorescence	10 particles/mL	60	[33]
–	Pb ^2+^	N	Cas12a	Fluorescence	0.053 nM	15	[34]
–	Na ^+^	N	Cas12a	Luminescence	0.37 nM	15	[35]
FRITCas13a	F ^-^	N	Cas13a	Fluorescence	1.7 μM	30	[36]

NASBA: nucleic acid sequence-based amplification. NA: nucleic acid. IL-6 indicates interleukin-6. VEGF: vascular endothelial growth factor. AFP: alpha fetoprotein. p-HBA: p-hydroxybenzoic acid.

## CRISPR-based Detection of Different Biomolecules

### Nucleic acid detection

**Figure 2:**
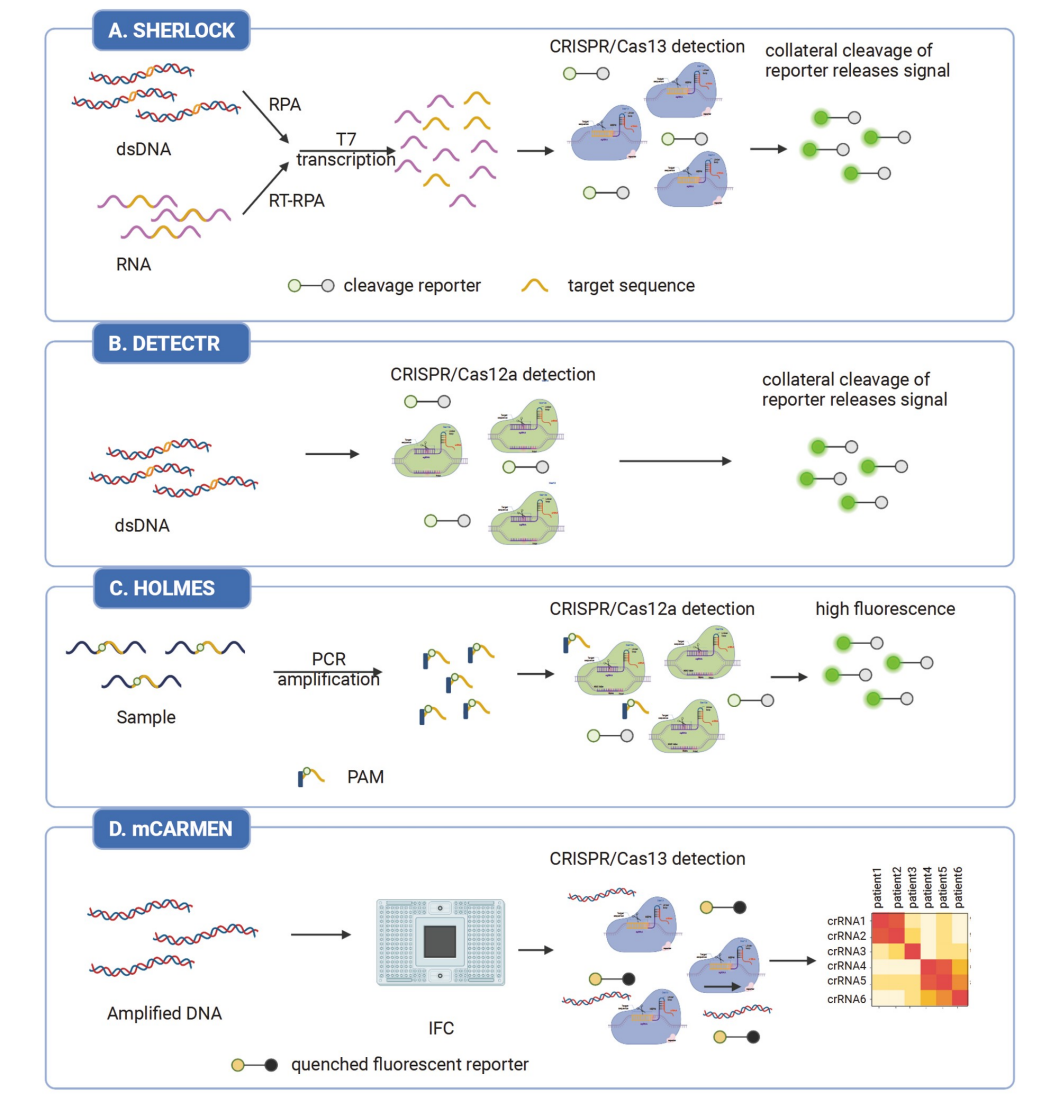
Figure 2 Several representative CRISPR-based nucleic acid detection technologies (A) SHERLOCK technology includes RPA pre-amplification, T7 transcription, and Cas13a detection. (B) DETECTR technology consists of RPA pre-amplification and Cas12a detection. (C) HOLMES technology is composed of PCR pre-amplification and Cas12a detection. (D) mCARMEN technology realizes the massively multiplexed viral detection using Cas13a with microfluidics. This figure was created with BioRender.com.

Nucleic acid detection is widely recognized as the gold standard in molecular diagnostics due to its high sensitivity and specificity. Emerging CRISPR-based nucleic acid detection technologies, such as SHERLOCK (specific high-sensitivity enzymatic reporter unlocking) [19], DETECTR (DNA endonuclease-targeted CRISPR transreporter) [3], and HOLMES (a one-hour low-cost multipurpose highly efficient system) [21], are regarded as the next generation of molecular diagnostic technologies ( Figure 2). The ability to specifically identify target sites and amplify signals is the core of the CRISPR system. Compared with RT-PCR, CRISPR-based detection technologies are easy to use and do not require lengthy thermal cycling, long turnaround time, or complex operations. During the pandemic of coronavirus disease 2019 (COVID-19), severe acute respiratory syndrome coronavirus 2 (SARS-CoV-2) detection methods based on SHERLOCK and DETECTR have been approved by the FDA for emergency use, which improve the detection efficiency and reduce the detection cost [ 37, 38].

With the development of CRISPR technologies, various nucleic acid detection methods have been developed and can be divided into several categories. First, high sample throughput methods, such as the microfluidic Combinatorial Arrayed Reactions for Multiplexed Evaluation of Nucleic acids (mCARMEN), can test hundreds of samples per day for multiple respiratory viruses and variants [ 22, 39]. Second, high multiplicity methods, such as SHERLOCKv2, which uses four orthogonal Cas proteins (LwaCas13a, CcaCas13b, LbaCas13a, and PsmCas13b) to achieve a 3.5-fold increase in sensitivity [20], require screening for compatible Cas proteins and an expensive multiple-channel fluorescence detector. Ansari *et al*. [40] utilized six CRISPRDx proteins of choice (FnCas9, enFnCas9, LwCas13a, LbCas12a, AaCas12b, and Cas14a) to *de novo* design gRNAs for SARS-CoV-2 variant detection. They offered queries for ready-to-use oligonucleotide sequences for validation on relevant samples. This method could greatly expand the portfolio of diagnostic applications, which contained a broad range of pathogenic and nonpathogenic conditions [40]. Microfluidic-assisted multiplex CRISPR detection, such as the Cas12a-based centrifugal microfluidic system to identify the Delta variant from wild-type SARS-CoV-2, is a better choice [ 8 , 41]. Third, one-pot detection methods, such as the one-pot visual RT-LAMP-CRISPR (opvCRISPR) method developed by Wang *et al*. [23], amplify RNA templates by RT-LAMP in the bottom of the tube and mix them with Cas12a reagents on the lid for detection. A similar method was designed by Chen *et al*. [42]. The nonspecific DNase activity of Cas14 has been harnessed to develop the DNA detection platform named DETECTR (DNA Endonuclease-Targeted CRISPR Trans Reporter)-Cas14 for diagnostic approaches in the human E3 ubiquitin-protein ligase ( *HERC2*) gene, which is responsible for iris color variability [12]. In comparison, Liu *et al*. [24] converted the one-pot assay to a digital quantification format called the microfluidics-enabled digital isothermal Cas13a assay (MEDICA), which takes advantage of droplet compartmentation. Without physical isolation, Ding *et al*. [25] also established a single reaction system named AIOD-CRISPR (All-In-One Dual CRISPR-Cas12a) for visual SARS-CoV-2 detection. Fourth, amplification-free detection methods, such as the amplification-free detection method for SARS-CoV-2 diagnosis developed by Fozouni *et al*. [43], commonly involve optimizing crRNAs and reporters, signal transducers, droplet-based digital CRISPR detection platforms, and cascade signal amplification [ 44‒ 46]. Fifth, quantification of targets can be achieved through methods such as droplet-based Cas12a or Cas13a assays in microdroplets [ 47, 48 ]. Finally, POCT methods, such as the Cas12a-based assay and portable smartphone-based fluorescence microscope device developed by Ning *et al* . [49] and the minimally instrumented SHERLOCK (miSHERLOCK) method developed by Puig *et al* . [26], are crucial for field-applicable detection during sudden pandemics.

Despite the great potential of CRISPR-based nucleic acid detection technologies, integrating all the above merits into one platform remains a challenge. PAM is still necessary for Cas proteins to recognize the target, which is a rate-limiting step for extensive applications [ 50, 51]. Additionally, the CRISPR system and auxiliary instruments are not yet as mature as PCR. Therefore, CRISPR-based nucleic acid detection technologies are unlikely to replace PCR in the short term.

### Protein detection

**Figure 3:**
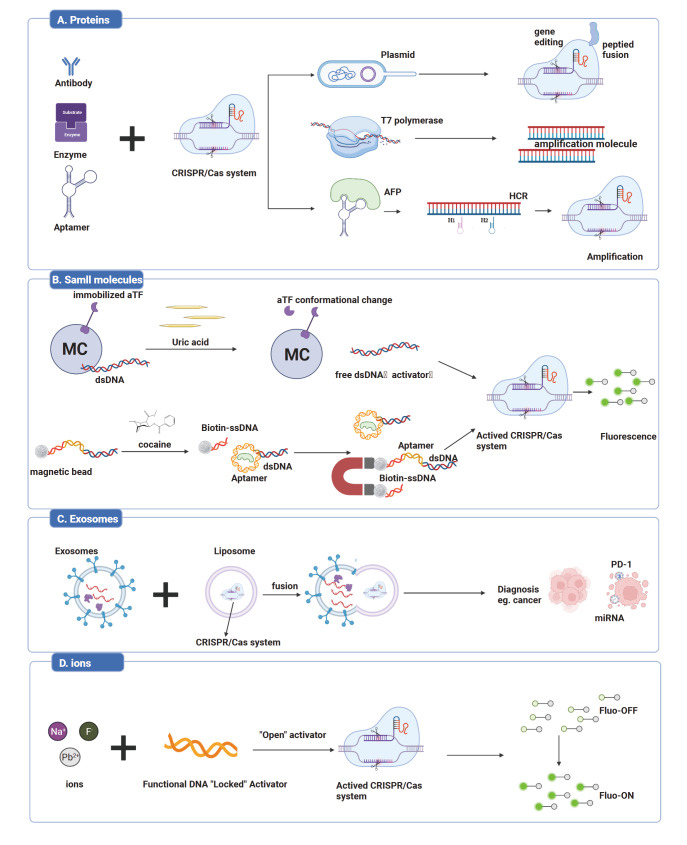
Figure 3 CRISPR-based detection of proteins, small molecules, exosomes, and ions Combined with functional DNA (fDNA) such as aTFs, aptamers and DNAzymes, CRISPR system could be applied for proteins (A), small molecules (B), exosomes (C) and metal ions (D) detection. This figure was created with BioRender.com.

Traditional protein detection methods such as immunoblotting, enzyme-linked immunosorbent assay (ELISA), and mass spectrometry (MS) are time-consuming and labor-intensive, and extra experimental steps such as purification can lead to false results [ 52, 53 ]. Although new methods have been developed, they often require specialized equipment and are costly [ 54‒ 56]. In recent years, CRISPR technology has been introduced to protein detection by means of a range of target recognition elements, including antibodies, enzymes and aptamers ( Figure 3A).

To improve sensitivity in detecting low concentrations of biomarkers, Baber *et al*. [57] developed a CRISPR-based peptide display technology called peptide immobilization by dCas9-mediated self-organization (PICASSO). This technology uses bespoke peptide libraries fused to catalytically inactive Cas9 (dCas9) and barcoded with unique single guide RNA (sgRNA) molecules to rapidly and multiplexly bind assays, enabling viral epitope mapping and multiplex diagnostics. Similarly, Tang *et al*. [27] developed an ultrasensitive CRISPR-Cas12a-based antibody detection (UCAD) assay to detect SARS-CoV-2 antibodies in clinical blood samples without complicated isolation steps. The technology converts SARS-CoV-2 antibodies in blood samples into predesigned nucleic acid sequences *in situ* and then utilizes RT-RPA and CRISPR-Cas12a *trans*-cleavage for ultrasensitive detection, achieving 10 ^4^ folds sensitivity of the commercial ELISA kit. Chen *et al*. [28] utilized T7 RNA polymerase and CRISPR-Cas13a to mediate double amplification output signals in a process called the CRISPR-Cas13a signal amplification linked immunosorbent assay (CLISA), which increased the sensitivity at least 10 ^2^ - *fold* compared to traditional ELISA. CLISA is adaptable to high-throughput and automation technologies, with superiority in detecting low abundance proteins. Furthermore, integrated with a 2D porphyrin metal-organic framework, CRISPR-Cas14 was used by Wu *et al*. [58] as a signal amplification tool to detect microcystin-LR (MC-LR), which is highly toxic and widely distributed in the environment. The new sensor was called the Cas14-pMOFs fluorescence sensor. The CRISPR/Cas14 system could greatly improve sensitivity, and the limit of detection (LOD) is 0.12 nM. This Cas14-pMOF fluorescence sensor is able to detect MC-LR in a range from 50 pg/mL to 1 μg/mL with an LOD of 19 pg/mL [58].

In addition to the SARS-CoV-2 antibodies and T7 RNA polymerase mentioned above, aptamers are also promising target recognition elements in protein detection. Liu *et al*. [59] developed a magnetic bead separation platform consisting of a switching aptamer-triggered hybridization chain reaction (SAT-HCR) and the CRISPR-Cas12a sensor for alpha fetoprotein (AFP), a marker of hepatoblastoma. The fluorescence intensity was proportional to the concentration of AFP in the range of 0.5–10 ^4^ ng/mL, showing greater sensitivity, lower cost, and higher selectivity compared to ELISA. Xing *et al*. [60] reported highly sensitive detection of tumor-derived extracellular vesicle (TEV) proteins using dual amplification of hybridization chain reaction (HCR) and CRISPR-Cas12a, including programmed death ligand 1, with an LOD as low as 10 ^2^ particles/μL, much more sensitive than ELISA. The comprehensive application of CRISPR technology and traditional methods greatly increases detection efficiency and sensitivity, opening new avenues for the timely discovery of protein biomarkers for different diseases.

### Small molecule detection

Small molecules are typically defined as chemical compounds with a molecular weight of less than 900 Daltons [61]. Due to their ability to diffuse across cell membranes and influence the function of biomolecules at various levels, rapid and accurate detection of small molecules is essential for assessing their impact on human health, the environment, and food safety [ 62‒ 65 ]. However, current methods for small molecule detection often involve complex procedures, long detection times, and low sensitivity. Therefore, CRISPR-based diagnostics represent a promising option for precise and fast small molecule detection ( Figure 3B).

Zhang’s group was the first to report on a CRISPR-Cas12a and allosteric transcription factor (aTF)-mediated small molecule detector called CaT-SMelor, which successfully detected nanomolar levels of uric acid and p-hydroxybenzoic acid [29]. They later coupled CRISPR-Cas12a and aptamers to develop CaT-SMelor 2.0 for the detection of diverse analytes, such as alpha fetoprotein (AFP) and cocaine [30]. Building on this work, many researchers have combined the CRISPR system with other technologies for small molecule detection. For example, Wang *et al*. [66] combined CRISPR-Cas12a with nanomaterials, such as upconversion nanoparticles (UCNPs) and metal-organic frameworks (MOFs), to develop a nanobiosensor for estradiol (E2) and prostate-specific antigen (PSA) detection. Samanta *et al*. [67] chose horseradish peroxidase (HRP) as the enzymatic reporter to develop a dual amplification sensing strategy, which couples analyte-induced Cas activation (Cas12a or Cas13a) to the release of HRP into solution for visual detection. To enable intelligent POCT (iPOCT), Zhao *et al*. [68] designed a Cas12a-powered portable smartphone-controlled reader for the detection of aflatoxin B1 (AFB1), benzo[a]pyrene (BaP), and capsaicin (CAP) in healthcare, environmental, and food settings. Li *et al*. [31] developed an isothermal proximity CRISPR Cas12a assay (iPCCA), where target recognition is achieved through proximity hybridization rather than crRNA binding. The performance of CRISPR/Cas14 in small molecule detection is unignorable. Hu *et al* . [69] optimized the element probe-based CRISPR/Cas14 detection platform to detect and trace aqueous ampicillin. In this method, the element probe ensured that Cas14 could prefer longer lengths in element probe cleavage with an LOD of 2.06 nM in complex matrix detection. In summary, CRISPR/Cas systems play an increasingly important role in small molecule detection.

To clarify the advantages and disadvantages of CRISPR-based methods, Table 2 was used to compare them with 4 other typical small molecule detection methods, including liquid chromatograph mass spectrometry (LC-MS/MS), chemiluminescence immunoassay, surface plasmon resonance (SPR), and aptamers. Briefly, LC-MS/MS is usually regarded as the gold standard for small molecule detection, but it requires complex sample preparation, expensive instruments and poisonous reagents. Although chemiluminescence immunoassays only require simple sample preparation and have high throughput, they are limited by sensitivity and specificity. SPR is another typical method, but it has low throughput and complex sensor modification. Aptamers need a complex screening process to obtain highly specific aptamers. In comparison, the CRISPR/cas system combined with other small molecule recognition elements could achieve higher sensitivity, specificity and throughput. Nevertheless, CRISPR technology is still in the fast development stage, and the system is immature and lacks standardization, so there is a shortage of commercial products.

**Table:** **Table 2** Comparison of typical small molecule detection methods and CRISPR-based detection

	LC-MS/MS		Chemiluminescence immunoassay		Surface plasmon resonance (SPR)		Aptamer		CRISPR/Cas
Principle	precise quantification of small molecule according to mass/charge ratio		combination of highly sensitive chemiluminescence with a highly specific immune response		identify a substance by its spectrum and determining its chemical composition and relative content		utilize oligonucleic nature and higher stability in harsh environments and longer shelf-life to detect small molecules		combination of the characteristics of CRISPR/Cas with other small molecules recognition elements
Advantages	high accuracy high specificity golden standard		high throughput simple sample preparation		simple operation high sensitivity less required samples real-time detection		high reproducibility low cost stable		high sensitivity high specificity high throughput
Disadvantages	time-consuming complex sample preparation expensive facility low throughput poisonous		limited sensitivity limited specificity limited accuracy		unstable sensor low throughput		complex screening process of valid aptamer limited to molecular size false positive cross reaction		in the development stage immature system lack standardization low commercialization
References	[ 70‒ 72]		[ 73‒ 75]		[ 76, 77]		[ 78‒ 80]		[ 29, 30, 66]

### Exosome detection

Extracellular vesicles (EVs) are tiny membrane-bound vesicles that are actively released by cells and have the potential to participate in maintaining homeostasis as well as contributing to various diseases [ 81, 82]. Exosomes, the smallest subset of EVs with an average diameter of ~100 nanometers, originate from endosomes and comprise mRNA, miRNA, DNA, protein, and lipids [83]. Through the transfer of RNA-containing exosomes to recipient cells, the protein mechanisms within the recipient cells are significantly influenced, contributing to both protection and pathologies within the body. Additionally, some identified RNA binding proteins in exosomes are also likely to play a role in the transfer process. As messengers in human health and disease, exosomes have an important function in metastatic spread, drug resistance, and angiogenesis in cancer [ 84, 85].

Because they are secreted by all cells and contain molecules from those cells, exosomes have potential use as biomarkers for diagnosis and prognosis. Exosomal miRNA, for instance, is critical in cancer diagnosis. Zhang *et al* . [32] developed a liposome-mediated membrane fusion strategy to transfect CRISPR-Cas13a into exosomes (MFS-CRISPR) to directly measure exosomal miRNAs in plasma. This strategy could detect exosomal miR-21 at concentrations as low as 1.2×10 ^3^ particles/mL. In this platform, RNA extraction, miRNA degradation, and contamination can be avoided. In addition, circulating miRNA could be used to induce strand displacement amplification without reverse transcription. Chi *et al*. [86] utilized the specificity of circulating miRNA and CRISPR/Cas14 to detect miR-21, a critical biomarker with overexpression in cholangiocarcinoma. The application of CRISPR-Cas14a could greatly reduce nonspecific amplification and improve the sensitivity by 2.86-fold compared to that using Cas12a. Another promising biomarker for monitoring cancer immunotherapy is PD-L1 in exosomes. He *et al*. [33] developed a new method named the aptamer-RPA-TMA-Cas13a assay (ARTCA) that utilized the collateral effect of Cas13a, the protein-binding aptamer, and isothermal amplification to detect exosomal PD-L1 proteins at an LOD of 10 particles/mL. Using ARTCA, the level of circulating exosomal PD-L1 significantly increases in patients with tumor progression. In addition to cancer diagnostics, the combination of CRISPR and exosomes is also used to detect infectious diseases. Ning *et al*. [87] developed an assay to accurately identify patients with COVID-19 by capturing exosomes from plasma and fusing them with reagent-loaded liposomes. Overall, CRISPR-based exosome detection has become an important research direction in disease diagnosis ( Figure 3C).

### Ion detection

Metal metal ions are environmental pollutants that can have significant impacts on human health, such as kidney damage, hypertension, and infertility [88]. To address this issue, accurate detection and quantification of metal ions is crucial. The CRISPR system, with its high base resolution and isothermal signal amplification, has opened a new era of biosensing applications. By combining CRISPR with functional DNA (fDNA), such as DNAzymes, CRISPR can be applied for metal ion detection ( Figure 3D) [34].

For cation detection, Li *et al*. [89] developed a CRISPR-Cas12a-based method for lead ion (Pb ^2+^) detection using a Pb ^2+^-specific DNAzyme GR-5. Only in the presence of Pb ^2+^ could the target sequence be released, subsequently activating Cas12a collateral activity. This approach allows for the detection of Pb ^2+^ at the picomolar level (~0.053 nM). In comparison, Xu *et al*. [90] reported a preamplification-free colorimetric strategy based on the assistance of MnO _2_ nanozymes and the CRISPR-Cas12a system for the detection of Pb ^2+^ [90]. Additionally, Tang’s group reported two methods for Na ^+^ detection. First, they designed a versatile CRISPR-Cas12a biosensor that combines fDNA-regulated target transduction, boosting upconversion luminescent resonance energy transfer (LRET), and biomimetic chip-assisted signal amplification [35]. This biosensor shows commendable specificity and sensitivity (~0.37 nM) toward Na ^+^. Second, they combined holographic optical tweezers with an energy-concentrating upconversion luminescence nanoparticle (UCNP)-triggered boosting LRET to develop another fDNA-regulated CRISPR-Cas12a biosensor [88]. Both signal readout methods demonstrate great potential for metal ion detection. CRISPR could be applied to heavier metal ions such as Cd ^2+^. Zhou *et al*. [91] developed a fluorometric biosensor named HARRY (highly sensitive aptamer-regulated Cas14 R-loop for bioanalysis). The diblock ssDNA could activate Cas14, and then Cas14a trans-cleavages the fluorescent reporter to amplify the fluorescence. Due to containing the aptamer sequence of specific targets, ssDNA-target could form an assembly via aptamer interaction to stop Cas14a activation. HARRY could detect Cd ^2+^ with detection limits at the low-nanomolar level, indicating improvement compared with Cas12a-based aptasensors in sensitivity and versatility [91].

For anion detection, Ma *et al*. [36] developed fluoride riboswitch-regulated transcription with a Cas13a tandem sensor called FRITCas13a to detect F ^-^ with an LOD of 1.7 μM. Using a portable fluorometer, FRITCas13a can complete the detection process within 30 min, demonstrating high potential for portable and real-time detection and quantification of fluoride in drinking water and other types of samples.

## Conclusions and Prospects

**Figure 4:**
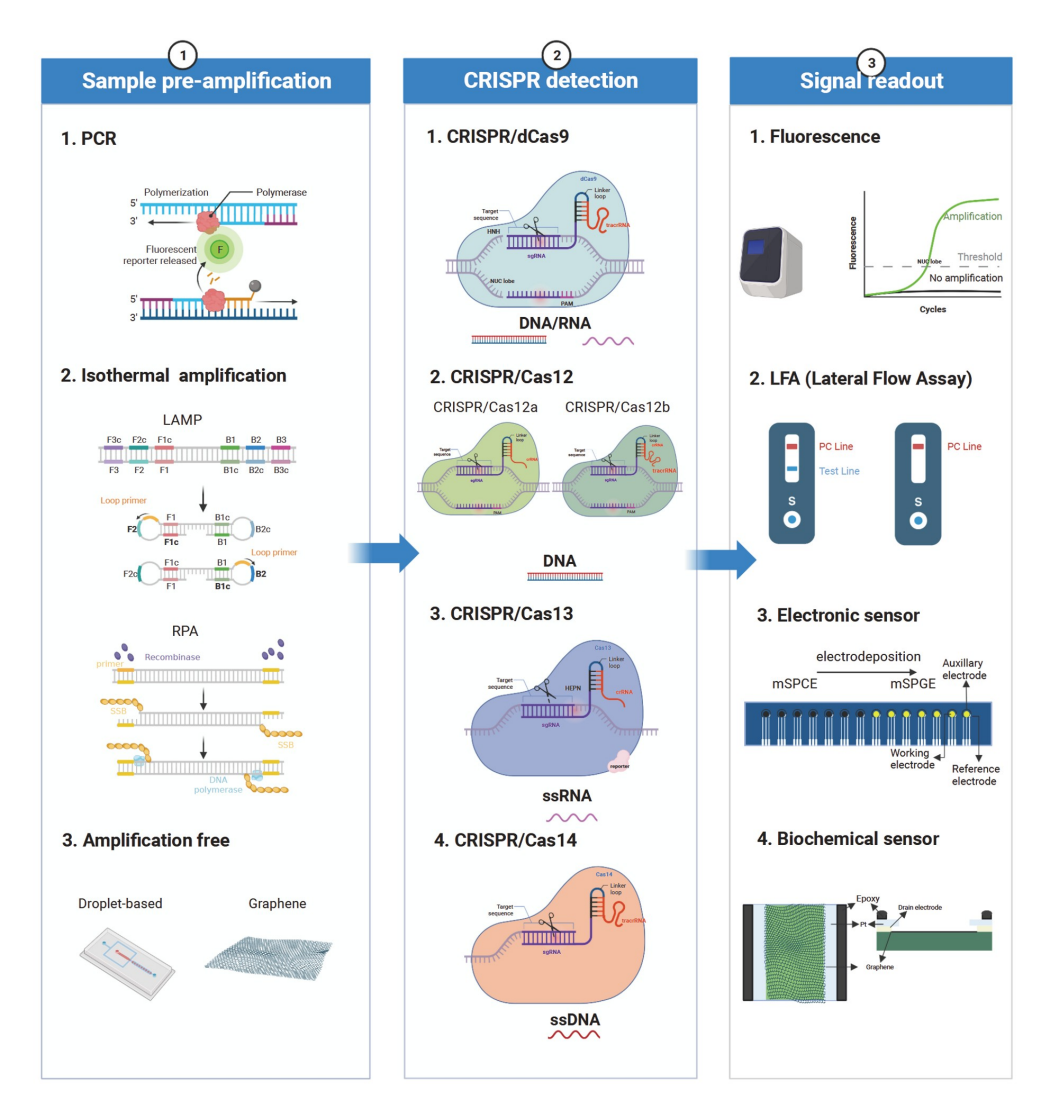
Figure 4 The general process of CRISPR-based detection technologies The whole detection process mainly consists of three parts: sample pre-amplification, CRISPR detection, and signal readout. The sample pre-amplification includes PCR, isothermal amplification, and amplification-free methods. CRISPR detection could choose the suitable Cas protein for detection, including dCas9, Cas12a, Cas12b, Cas13, Cas14, etc. The signal readout methods mainly includes fluorescence detection, LFA, electronic sensor, and biochemical sensor. This figure was created with BioRender.com.

CRISPR technologies have demonstrated remarkable potential in molecular diagnostics by detecting different biomolecules, including nucleic acids, proteins, small molecules, exosomes, and metal ions. To better illustrate the versatility of CRISPR technologies, we have created a figure to summarize their common pattern in molecular diagnostics ( Figure 4). Briefly, it consists of three parts: sample preamplification, CRISPR detection, and signal readout.

Sample preamplification usually involves PCR and isothermal amplification (LAMP or RPA) to enhance sensitivity. More importantly, for some detection sites, they need to introduce the PAM sequence into the template by means of primers because PAM is necessary for CRISPR/Cas/crRNA recognition. However, the two-step process of sample preamplification and CRISPR detection can make the operation more complicated and increase the possibility of aerosol contamination, which easily causes false positive results. Therefore, researchers are developing one-pot or amplification-free methods. One-pot methods integrate preamplification reagents into the CRISPR detection system by optimizing the reaction buffer [25] or designing the reaction process. The optimization of the reaction buffer is a time-consuming process. In contrast, physical isolation, represented by microfluidics, is a common strategy. For example, Li *et al* . [41] used an automated Cas12a-microfluidic system for rapid differential diagnosis of the B.1.617.2 (delta) variant of SARS-CoV-2. Apart from this, amplification-free methods eliminate the limitations of preamplification and enhance sensitivity through four aspects, including optimizing crRNAs and reporters, droplet-based digital CRISPR detection platforms, signal transducers, and cascade signal amplification [ 43‒ 45]. Combining two or three crRNAs can synergistically activate Cas proteins, resulting in more released fluorescence and thereby improving the LOD. Digital CRISPR methods isolating the individual reaction in each microunit can provide a novel capability of quantification and improve the sensitivity. For example, Shinoda *et al* . [46] developed a CRISPR/Cas-based amplification-free digital RNA detection (SATORI) method with the combination of CRISPR/Cas13 and microchamber-array technologies. Signal transducers can alter fluorescence detection to either electrochemical or surface-enhanced Raman scattering (SERS) signal output. The cascade reaction focuses on protease-based or nucleic acid circuit-based signal amplification systems. These strategies provide feasible ideas to enhance sensitivity without preamplification.

After sample preamplification, CRISPR detection offers a range of Cas proteins, including Cas9, dCas9, Cas12a, Cas12b, Cas13a, and Cas14a. Researchers can choose the appropriate Cas protein based on its characteristics and the downstream technologies they intend to integrate. For example, Cas9, Cas12a, Cas12b, and Cas14a directly recognize DNA as the template, while Cas13a directly recognizes RNA. Moreover, Cas12a, Cas12b, Cas13a, and Cas14a have collateral activity and can nonspecifically cleave fluorescent reporters, while Cas9 and dCas9 do not. Different Cas proteins have different sgRNA structures, including spacer and direct repeat regions, so the rules for designing the sgRNA also differ. Choosing the appropriate Cas protein is crucial for establishing CRISPR-based detection technologies.

Finally, various signal readout methods, including fluorescence, LFA (lateral flow assay), electronic sensors, and biochemical sensors, have been developed for different scenarios. Fluorescence is widely applied in CRISPR detection because of its straightforward readout, low cost and easy synthesis, but it has a relatively high background signal, which can sometimes cause false positive interpretation. Although LFA is convenient and fast, it is often limited by sensitivity and disturbed by external factors. In comparison, electrochemical sensors can simultaneously realize high sensitivity and specificity through a slight change in current. There is no denying that the fabrication of electrochemical sensors is slightly complex, and it has high requirements for the choice of materials and modification. Overall, different sample preamplification methods and postdetection signal readouts contribute to improving the sensitivity and scalability of their applications. The key to effectively combining these two parts with the CRISPR system is optimization.

As an emerging technology, the development trend of CRISPR also fits the hype cycle. It underwent the stage of technology trigger, peak of inflated expectations and trough of disillusionment, and now it is in the stage of slope of enlightenment. No method is perfect. To clarify the strengths and weaknesses, we have created a table comparing the current mainstream CRISPR-based detection methods ( Table 1). Through the comparison from different aspects, we have also summarized the room for optimization and improvement of CRISPR-based diagnostics. First, standardization needs to be further enhanced for the definition of trans-cleavage enzymatic units of Cas proteins, the components of reaction buffer and the criteria of crRNA design. Many factors, including different batches of proteins, ion levels, temperature, and pH, can influence the enzyme activity of Cas proteins. The Cas proteins used in the present system are usually quantitated in concentration instead of enzymatic units, so the *trans*-cleavage activities may vary from different commercial providers. Therefore, Lv *et al* . [92] first defined the Cas12 trans-cleavage units to facilitate CRISPR diagnostics. The reaction buffer commonly used for the CRISPR system is NEBuffer 3.0 or NEBuffer 2.1, and no study has developed a specialized reaction buffer for different Cas proteins. More importantly, there is still a lack of a commercial website or software to design crRNAs, and people can only design them through some general rules, which will require considerable time for screening.

Second, exploring new Cas proteins or engineering existing ones is essential since current Cas proteins still have some drawbacks in characteristics, such as PAM dependence, narrow reaction temperature range, and large molecular weight. Many researchers are dedicated to finding Cas proteins without the limits of PAM because for different template detection, we usually need to find the PAM sequence first, but sometimes the target sequence does not have PAM. One approach we can take is to introduce the PAM through nucleic acid amplification, which will make the entire system more complex. Moreover, the reaction temperature range is another limiting factor for the development of CRISPR technologies. At present, the reaction temperature of Cas12a, Cas13a and Cas14a is approximately 37°C, and Cas12b can react at up to 60°C. There is still a lack of more thermophilic Cas proteins for diagnostics, which limits their combination with other technologies. The molecular weight of current CRISPR-based diagnostics (CRISPR-Dx) enzymes is more than 100 kDa, which puts forward higher requirements for protein purification and reaction system construction. To some extent, exploring smaller Cas proteins will speed the process of CRISPR-Dx development.

Third, to satisfy the requirements of multiscenario detection, researchers aim to integrate all the characteristics of high sample throughput, multiplicity, portability, target quantification, one-pot, and amplification-free detection into a POCT device. Establishing a truly integrated detection platform is a complex multidisciplinary problem requiring the combinatorial development of upstream sample pretreatment, amplification, and downstream detection technologies. For high sample throughput, CRISPR-Dx can be combined with the current high-throughput platform, such as 96-well or 384-well plate workstations, to realize massive sample loading and signal readout. For multiplicity and portability, microfluidics is the first choice to integrate because it has many microwells or microchannels to isolate different reaction systems. Meanwhile, supporting equipment based on microfluidics can be easily designed into a portable form. For target quantification, digital CRISPR (microdroplets or microchips) and standard curves are two different methods. The former is absolute quantification, and the latter is relative quantification. To realize POCT, freeze-drying of the CRISPR reagents must be taken into consideration to eliminate the need for a cold chain. The operators only need to add the samples and hydrated solutions to mix uniformly, and then the whole reaction process can be accomplished in an automatic and miniaturized instrument. Overall, we believe the cost, affordability, and robustness will determine the success of these CRISPR-based detection technologies in the long run.

## References

[1] Pan MM, Wang YF, Wang L, Yu X, Xu L (2021). Recent advances in visual detection for cancer biomarkers and infectious pathogens. J Mater Chem B.

[2] Li L, Shen G, Wu M, Jiang J, Xia Q, Lin P (2022). CRISPR-Cas-mediated diagnostics. Trends Biotechnol.

[3] Chen JS, Ma E, Harrington LB, Da Costa M, Tian X, Palefsky JM, Doudna JA (2018). CRISPR-Cas12a target binding unleashes indiscriminate single-stranded DNase activity. Science.

[4] Su Y, Yuan D, Chen DG, Ng RH, Wang K, Choi J, Li S (2022). Multiple early factors anticipate post-acute COVID-19 sequelae. Cell.

[5] Myhrvold C, Freije CA, Gootenberg JS, Abudayyeh OO, Metsky HC, Durbin AF, Kellner MJ (2018). Field-deployable viral diagnostics using CRISPR-Cas13. Science.

[6] Pickar-Oliver A, Gersbach CA (2019). The next generation of CRISPR-Cas technologies and applications. Nat Rev Mol Cell Biol.

[7] Pardee K, Green AA, Takahashi MK, Braff D, Lambert G, Lee JW, Ferrante T (2016). Rapid, low-cost detection of zika virus using programmable biomolecular components. Cell.

[8] Abudayyeh OO, Gootenberg JS, Konermann S, Joung J, Slaymaker IM, Cox DBT, Shmakov S (2016). C2c2 is a single-component programmable RNA-guided RNA-targeting CRISPR effector. Science.

[9] East-Seletsky A, O′Connell MR, Knight SC, Burstein D, Cate JHD, Tjian R, Doudna JA (2016). Two distinct RNase activities of CRISPR-C2c2 enable guide-RNA processing and RNA detection. Nature.

[10] Li SY, Cheng QX, Liu JK, Nie XQ, Zhao GP, Wang J (2018). CRISPR-Cas12a has both cis- and trans-cleavage activities on single-stranded DNA. Cell Res.

[11] Li L, Li S, Wu N, Wu J, Wang G, Zhao G, Wang J (2019). HOLMESv2: a CRISPR-Cas12b-assisted platform for nucleic acid detection and DNA methylation quantitation. ACS Synth Biol.

[12] Harrington LB, Burstein D, Chen JS, Paez-Espino D, Ma E, Witte IP, Cofsky JC (2018). Programmed DNA destruction by miniature CRISPR-Cas14 enzymes. Science.

[13] Zetsche B, Gootenberg JS, Abudayyeh OO, Slaymaker IM, Makarova KS, Essletzbichler P, Volz SE (2015). Cpf1 is a single RNA-guided endonuclease of a class 2 CRISPR-cas system. Cell.

[14] Lu S, Tong X, Han Y, Zhang K, Zhang Y, Chen Q, Duan J (2022). Fast and sensitive detection of SARS-CoV-2 RNA using suboptimal protospacer adjacent motifs for Cas12a. Nat Biomed Eng.

[15] Lee H, Choi J, Jeong E, Baek S, Kim HC, Chae JH, Koh Y (2018). dCas9-mediated nanoelectrokinetic direct detection of target gene for liquid biopsy. Nano Lett.

[16] Yang W, Restrepo-Pérez L, Bengtson M, Heerema SJ, Birnie A, van der Torre J, Dekker C (2018). Detection of CRISPR-dcs9 on DNA with solid-state nanopores. Nano Lett.

[17] Moon J, Kwon HJ, Yong D, Lee IC, Kim H, Kang H, Lim EK (2020). Colorimetric detection of SARS-CoV-2 and drug-resistant pH1N1 using CRISPR/dCas9. ACS Sens.

[18] Hajian R, Balderston S, Tran T, deBoer T, Etienne J, Sandhu M, Wauford NA (2019). Detection of unamplified target genes via CRISPR-Cas9 immobilized on a graphene field-effect transistor. Nat Biomed Eng.

[19] Gootenberg JS, Abudayyeh OO, Lee JW, Essletzbichler P, Dy AJ, Joung J, Verdine V (2017). Nucleic acid detection with CRISPR-Cas13a/C2c2. Science.

[20] Gootenberg JS, Abudayyeh OO, Kellner MJ, Joung J, Collins JJ, Zhang F (2018). Multiplexed and portable nucleic acid detection platform with Cas13, Cas12a, and Csm6. Science.

[21] Li SY, Cheng QX, Wang JM, Li XY, Zhang ZL, Gao S, Cao RB (2018). CRISPR-Cas12a-assisted nucleic acid detection. Cell Discov.

[22] Welch NL, Zhu M, Hua C, Weller J, Mirhashemi ME, Nguyen TG, Mantena S (2022). Multiplexed CRISPR-based microfluidic platform for clinical testing of respiratory viruses and identification of SARS-CoV-2 variants. Nat Med.

[23] Wang R, Qian C, Pang Y, Li M, Yang Y, Ma H, Zhao M (2021). opvCRISPR: One-pot visual RT-LAMP-CRISPR platform for SARS-CoV2-2 detection. Biosens Bioelectron.

[24] Liu FX, Cui JQ, Park H, Chan KW, Leung T, Tang BZ, Yao S (2022). Isothermal background-free nucleic acid quantification by a one-pot cas13a assay using droplet microfluidics. Anal Chem.

[25] Ding X, Yin K, Li Z, Lalla RV, Ballesteros E, Sfeir MM, Liu C (2020). Ultrasensitive and visual detection of SARS-CoV-2 using all-in-one dual CRISPR-Cas12a assay. Nat Commun.

[26] de Puig H, Lee RA, Najjar D, Tan X, Soekensen LR, Angenent-Mari NM, Donghia NM (2021). Minimally instrumented SHERLOCK (miSHERLOCK) for CRISPR-based point-of-care diagnosis of SARS-CoV-2 and emerging variants. Sci Adv.

[27] Tang Y, Song T, Gao L, Yin S, Ma M, Tan Y, Wu L (2022). A CRISPR-based ultrasensitive assay detects attomolar concentrations of SARS-CoV-2 antibodies in clinical samples. Nat Commun.

[28] Chen Q, Tian T, Xiong E, Wang P, Zhou X (2020). CRISPR/Cas13a signal amplification linked immunosorbent assay for femtomolar protein detection. Anal Chem.

[29] Liang M, Li Z, Wang W, Liu J, Liu L, Zhu G, Karthik L (2019). A CRISPR-Cas12a-derived biosensing platform for the highly sensitive detection of diverse small molecules. Nat Commun.

[30] Zhao X, Li S, Liu G, Wang Z, Yang Z, Zhang Q, Liang M (2021). A versatile biosensing platform coupling CRISPR-Cas12a and aptamers for detection of diverse analytes. Sci Bull.

[31] Li Y, Mansour H, Watson CJF, Tang Y, MacNeil AJ, Li F (2021). Amplified detection of nucleic acids and proteins using an isothermal proximity CRISPR Cas12a assay. Chem Sci.

[32] Zhang J, Guan M, Ma C, Liu Y, Lv M, Zhang Z, Gao H (2023). Highly effective detection of exosomal miRNAs in plasma using liposome-mediated transfection CRISPR/Cas13a. ACS Sens.

[33] He Y, Wu Y, Wang Y, Wang X, Xing S, Li H, Guo S,
*et al*. Applying CRISPR/Cas13 to construct exosomal PD‐L1 ultrasensitive biosensors for dynamic monitoring of tumor progression in immunotherapy.
Advanced Therapeutics 2020, 3: 10.1002/adtp.202000093. https://doi.org/10.1002/adtp.202000093.

[34] Xiong Y, Zhang J, Yang Z, Mou Q, Ma Y, Xiong Y, Lu Y (2020). Functional DNA regulated CRISPR-Cas12a sensors for point-of-care diagnostics of non-nucleic-acid targets. J Am Chem Soc.

[35] Li CY, Zheng B, Liu YH, Gao JL, Zheng MQ, Pang DW, Tang HW (2020). A boosting upconversion luminescent resonance energy transfer and biomimetic periodic chip integrated CRISPR/Cas12a biosensor for functional DNA regulated transduction of non-nucleic acid targets. Biosens Bioelectron.

[36] Ma Y, Mou Q, Yan P, Yang Z, Xiong Y, Yan D, Zhang C (2021). A highly sensitive and selective fluoride sensor based on a riboswitch-regulated transcription coupled with CRISPR-Cas13a tandem reaction. Chem Sci.

[37] Palaz F, Kalkan AK, Tozluyurt A, Ozsoz M (2021). CRISPR-based tools: alternative methods for the diagnosis of COVID-19. Clin Biochem.

[38] Mustafa MI, Makhawi AM. SHERLOCK and DETECTR: CRISPR-Cas systems as potential rapid diagnostic tools for emerging infectious diseases.
JClinMicrobiol 2021, 59: e00745-20. https://doi.org/10.1128/JCM.00745-20.

[39] Ackerman CM, Myhrvold C, Thakku SG, Freije CA, Metsky HC, Yang DK, Ye SH (2020). Massively multiplexed nucleic acid detection with Cas13. Nature.

[40] Ansari AH, Kumar M, Sarkar S, Maiti S, Chakraborty D (2023). CriSNPr, a single interface for the curated and de novo design of gRNAs for CRISPR diagnostics using diverse Cas systems. eLife.

[41] Li P, Zhang J, Lin Q, Kong J, Fang X (2021). Rapid differential diagnosis of the B.1.617.2 (delta) variant of SARS-CoV-2 using an automated Cas12a-microfluidic system. Chem Commun.

[42] Chen Y, Qian S, Yu X, Wu J, Xu J (2023). Microfluidics: the propellant of CRISPR-based nucleic acid detection. Trends Biotechnol.

[43] Fozouni P, Son S, Díaz de León Derby M, Knott GJ, Gray CN, D’Ambrosio MV, Zhao C (2021). Amplification-free detection of SARS-CoV-2 with CRISPR-Cas13a and mobile phone microscopy. Cell.

[44] Li H, Xie Y, Chen F, Bai H, Xiu L, Zhou X, Guo X (2023). Amplification-free CRISPR/Cas detection technology: challenges, strategies, and perspectives. Chem Soc Rev.

[45] Zhang J, Lv H, Li L, Chen M, Gu D, Wang J, Xu Y (2021). Recent improvements in CRISPR-Based amplification-free pathogen detection. Front Microbiol.

[46] Shinoda H, Taguchi Y, Nakagawa R, Makino A, Okazaki S, Nakano M, Muramoto Y (2021). Amplification-free RNA detection with CRISPR-Cas13. Commun Biol.

[47] Yue H, Shu B, Tian T, Xiong E, Huang M, Zhu D, Sun J (2021). Droplet cas12a assay enables dna quantification from unamplified samples at the single-molecule level. Nano Lett.

[48] Tian T, Shu B, Jiang Y, Ye M, Liu L, Guo Z, Han Z (2021). An ultralocalized cas13a assay enables universal and nucleic acid amplification-free single-molecule RNA diagnostics. ACS Nano.

[49] Ning B, Yu T, Zhang S, Huang Z, Tian D, Lin Z, Niu A (2021). A smartphone-read ultrasensitive and quantitative saliva test for COVID-19. Sci Adv.

[50] Jiang F, Doudna JA (2017). CRISPR–Cas9 structures and mechanisms. Annu Rev Biophys.

[51] Lee H, Sashital DG (2022). Creating memories: molecular mechanisms of CRISPR adaptation. Trends Biochem Sci.

[52] Palaz F, Kalkan AK, Can Ö, Demir AN, Tozluyurt A, Özcan A, Ozsoz M (2021). CRISPR-Cas13 system as a promising and versatile tool for cancer diagnosis, therapy, and research. ACS Synth Biol.

[53] Tang XZE, Tan SX, Hoon S, Yeo GW (2022). Pre-existing adaptive immunity to the RNA-editing enzyme Cas13d in humans. Nat Med.

[54] Shao H, Im H, Castro CM, Breakefield X, Weissleder R, Lee H (2018). New technologies for analysis of extracellular vesicles. Chem Rev.

[55] Chen Z, Chen JJ, Fan R (2019). Single-cell protein secretion detection and profiling. Annu Rev Anal Chem.

[56] Lou B, Liu Y, Shi M, Chen J, Li K, Tan Y, Chen L (2022). Aptamer-based biosensors for virus protein detection. TrAC Trends Anal Chem.

[57] Barber KW, Shrock E, Elledge SJ (2021). CRISPR-based peptide library display and programmable microarray self-assembly for rapid quantitative protein binding assays. Mol Cell.

[58] Wu P, Ye X, Wang D, Gong F, Wei X, Xiang S, Zhang J (2022). A novel CRISPR/Cas14a system integrated with 2D porphyrin metal-organic framework for microcystin-LR determination through a homogeneous competitive reaction. J Hazard Mater.

[59] Liu Y, Chen Y, Zhang Y, Zhong Q, Zhu X, Wu Q (2022). A functionalized magnetic nanoparticle regulated CRISPR-Cas12a sensor for the ultrasensitive detection of alpha-fetoprotein. Analyst.

[60] Xing S, Lu Z, Huang Q, Li H, Wang Y, Lai Y, He Y (2020). An ultrasensitive hybridization chain reaction-amplified CRISPR-Cas12a aptasensor for extracellular vesicle surface protein quantification. Theranostics.

[61] Wally V, Reisenberger M, Kitzmüller S, Laimer M (2020). Small molecule drug development for rare genodermatoses – evaluation of the current status in epidermolysis bullosa. Orphanet J Rare Dis.

[62] Shin J, Miller M, Wang Y‐ (2022). Recent advances in CRISPR‐based systems for the detection of foodborne pathogens. Comp Rev Food Sci Food Safe.

[63] Mao Z, Chen R, Wang X, Zhou Z, Peng Y, Li S, Han D (2022). CRISPR/Cas12a-based technology: a powerful tool for biosensing in food safety. Trends Food Sci Tech.

[64] Huang Z, Fang J, Zhou M, Gong Z, Xiang T (2022). CRISPR-Cas13: a new technology for the rapid detection of pathogenic microorganisms. Front Microbiol.

[65] Nguyen PQ, Soenksen LR, Donghia NM, Angenent-Mari NM, de Puig H, Huang A, Lee R (2021). Wearable materials with embedded synthetic biology sensors for biomolecule detection. Nat Biotechnol.

[66] Wang Y, Peng Y, Li S, Han D, Ren S, Qin K, Zhou H (2023). The development of a fluorescence/colorimetric biosensor based on the cleavage activity of CRISPR-Cas12a for the detection of non-nucleic acid targets. J Hazard Mater.

[67] Samanta D, Ebrahimi SB, Ramani N, Mirkin CA (2022). Enhancing CRISPR-Cas-mediated detection of nucleic acid and non-nucleic acid targets using enzyme-labeled reporters. J Am Chem Soc.

[68] Zhao Y, Wu W, Tang X, Zhang Q, Mao J, Yu L, Li P (2023). A universal CRISPR/Cas12a-powered intelligent point-of-care testing platform for multiple small molecules in the healthcare, environment, and food. Biosens Bioelectron.

[69] Hu J, Zhou J, Liu R, Lv Y (2021). Element probe based CRISPR/Cas14 bioassay for non-nucleic-acid targets. Chem Commun.

[70] Chen F, Cheng Z, Peng Y, Wang Z, Huang C, Liu D, Wang B (2021). A liquid chromatography-tandem mass spectrometry (LC-MS/MS)-based assay for simultaneous quantification of aldosterone, renin activity, and angiotensin II in human plasma. J Chromatography B.

[71] Wise SA, Camara JE, Sempos CT, Lukas P, Le Goff C, Peeters S, Burdette CQ (2021). Vitamin D standardization program (VDSP) intralaboratory study for the assessment of 25-hydroxyvitamin D assay variability and bias. J Steroid Biochem Mol Biol.

[72] Tolan NV, Yoon EJ, Brady AR, Horowitz GL (2018). Price of high-throughput 25-hydroxyvitamin d immunoassays: frequency of inaccurate results. J Appl Lab Med.

[73] Rosner W, Hankinson SE, Sluss PM, Vesper HW, Wierman ME (2013). Challenges to the measurement of estradiol: an endocrine society position statement. J Clin Endocrinol Metab.

[74] Naruse M, Katabami T, Shibata H, Sone M, Takahashi K, Tanabe A, Izawa S (2022). Japan Endocrine Society clinical practice guideline for the diagnosis and management of primary aldosteronism 2021. Endocr J.

[75] Cabello MC, Bartoloni FH, Bastos EL, Baader WJ (2023). The molecular basis of organic chemiluminescence. Biosensors.

[76] Fu E, Chinowsky T, Nelson K, Johnston K, Edwards T, Helton K, Grow M (2007). SPR imaging-based salivary diagnostics system for the detection of small molecule analytes. Ann New York Acad Sci.

[77] Olaru A, Bala C, Jaffrezic-Renault N, Aboul-Enein HY (2015). Surface plasmon resonance (SPR) biosensors in pharmaceutical analysis. Crit Rev Anal Chem.

[78] Prante M, Segal E, Scheper T, Bahnemann J, Walter J (2020). Aptasensors for point-of-care detection of small molecules. Biosensors.

[79] Dong Y, Zhang T, Lin X, Feng J, Luo F, Gao H, Wu Y (2020). Graphene/aptamer probes for small molecule detection: from
*in vitro* test to
*in situ* imaging. Microchim Acta.

[80] Yu H, Alkhamis O, Canoura J, Liu Y, Xiao Y (2021). Advances and challenges in small‐molecule DNA aptamer isolation, characterization, and sensor development. Angew Chem Int Ed.

[81] Kalluri R, LeBleu VS (2020). The biology, function, and biomedical applications of exosomes. Science.

[82] Evguenieva‐Hackenberg E, Hou L, Glaeser S, Klug G (2014). Structure and function of the archaeal exosome. Wiley Interdiscip Rev RNA.

[83] van Niel G, D′Angelo G, Raposo G (2018). Shedding light on the cell biology of extracellular vesicles. Nat Rev Mol Cell Biol.

[84] Fang Y, Ni J, Wang YS, Zhao Y, Jiang LQ, Chen C, Zhang RD (2023). Exosomes as biomarkers and therapeutic delivery for autoimmune diseases: Opportunities and challenges. AutoImmun Rev.

[85] Yu W, Hurley J, Roberts D, Chakrabortty SK, Enderle D, Noerholm M, Breakefield XO (2021). Exosome-based liquid biopsies in cancer: opportunities and challenges. Ann Oncol.

[86] Chi Z, Wu Y, Chen L, Yang H, Khan MR, Busquets R, Huang N (2022). CRISPR-Cas14a-integrated strand displacement amplification for rapid and isothermal detection of cholangiocarcinoma associated circulating microRNAs. Anal Chim Acta.

[87] Ning B, Huang Z, Youngquist BM, Scott JW, Niu A, Bojanowski CM, Zwezdaryk KJ (2021). Liposome-mediated detection of SARS-CoV-2 RNA-positive extracellular vesicles in plasma. Nat Nanotechnol.

[88] Li CY, Zheng B, Li JT, Gao J, Liu YH, Pang DW, Tang HW (2021). Holographic optical tweezers and boosting upconversion luminescent resonance energy transfer combined clustered regularly interspaced short palindromic repeats (CRISPR)/Cas12a biosensors. ACS Nano.

[89] Li J, Yang S, Zuo C, Dai L, Guo Y, Xie G (2020). Applying CRISPR-Cas12a as a signal amplifier to construct biosensors for Non-DNA targets in ultralow concentrations. ACS Sens.

[90] Xu S, Wang S, Guo L, Tong Y, Wu L, Huang X (2023). Nanozyme-catalysed CRISPR-Cas12a system for the preamplification-free colorimetric detection of lead ion. Anal Chim Acta.

[91] Zhou B, Yang R, Sohail M, Kong X, Zhang X, Fu N, Li B (2023). CRISPR/Cas14 provides a promising platform in facile and versatile aptasensing with improved sensitivity. Talanta.

[92] Lv H, Wang J, Zhang J, Chen Y, Yin L, Jin D, Gu D (2021). Definition of CRISPR Cas12a trans-cleavage units to facilitate crispr diagnostics. Front Microbiol.

